# Merkel Cell Carcinoma: A Complete Response to Avelumab Immunotherapy

**DOI:** 10.1002/ccr3.71768

**Published:** 2026-02-02

**Authors:** Eamonn Byrne, Mel Corbett, Eoin Conlon, Fergal O'Duffy

**Affiliations:** ^1^ Department of Otolaryngology Saint Vincent's University Hospital Dublin Ireland; ^2^ University College Dublin Head and Neck Oncology Group University College Dublin Dublin Ireland; ^3^ Department of Head and Neck Surgery Mater Misericordiae University Hospital Dublin Ireland

**Keywords:** immunotherapy, Merkel cell carcinoma, oncology, otolaryngology

## Abstract

Merkel cell carcinoma is an aggressive form of cancer with poor prognosis, particularly for individuals deemed unsuitable for surgical resection. With new immunotherapy agents being used alone or as an adjuvant treatment, improving long term outcomes are being seen, even for those deemed to be treated with palliative intent.

## 1 Introduction

Merkel cell carcinoma (MCC) is a rare neuroendocrine cutaneous malignancy [1]. It typically appears in sun‐exposed areas of the skin.^2^ Tumors arise due to carcinogenesis associated with the Merkel cell polyomavirus or chronic ultraviolet (UV) exposure [1, 2]. MCC is an aggressive tumor type, with mortality rates 15% higher than cutaneous melanoma [3]. Five‐year overall survival in MCC ranges from 48%–63%, with this falling to 14% in distant metastatic disease, defined as stage IV as per the AJCC 8th edition staging system [4, 5]. Distant dissemination occurs in 33% of MCC patients, with lymph nodes being the most common site, followed by liver and then bone marrow metastases [6].

For primary tumors, wide local excision followed by adjuvant radiotherapy is the first line treatment. Surgical excision is the treatment of choice for local recurrence or lymph node metastases. In the case of advanced stage IV MCC systemic treatment with anti‐programmed death‐1 (PD‐1) or anti‐programmed death‐ligand 1 (PD‐L1) immunotherapy should be offered to patients as first line treatment [7].

In 2017, Avelumab, an anti‐PD‐L1 monoclonal antibody, was the first immunotherapy approved for treatment of advanced or metastatic MCC in the US and Europe. Response to avelumab has been shown to be similar irrespective of PD‐L1 expression in tumor cells and so testing is not a prerequisite before beginning treatment with the immunotherapy [8].

The use of immune checkpoint inhibitors as an adjuvant agent is also something which is being evaluated in phase II and III clinical trials currently [9]. A number of these trials are examining immunotherapy in conjunction with radiation combinations as a treatment option for advanced MCC, similar to the case we present here [10].

## 2 Case Presentation

An 85 old man presented to the tertiary head and neck oncology unit outpatient clinic with bleeding from a new left sided neck mass at levels II‐V first noted five months previously. He had no other cervical lymphadenopathy clinically. He had lost 5 kilograms in the previous six months. His Eastern Cooperative Oncology Group performance score (ECOG) was judged to be 1 on presentation [11]. His background history was significant for superficial basal cell carcinoma in the lower back region for which he underwent wide local excision 8 years prior.

## 3 Investigations

**FIGURE 1:**
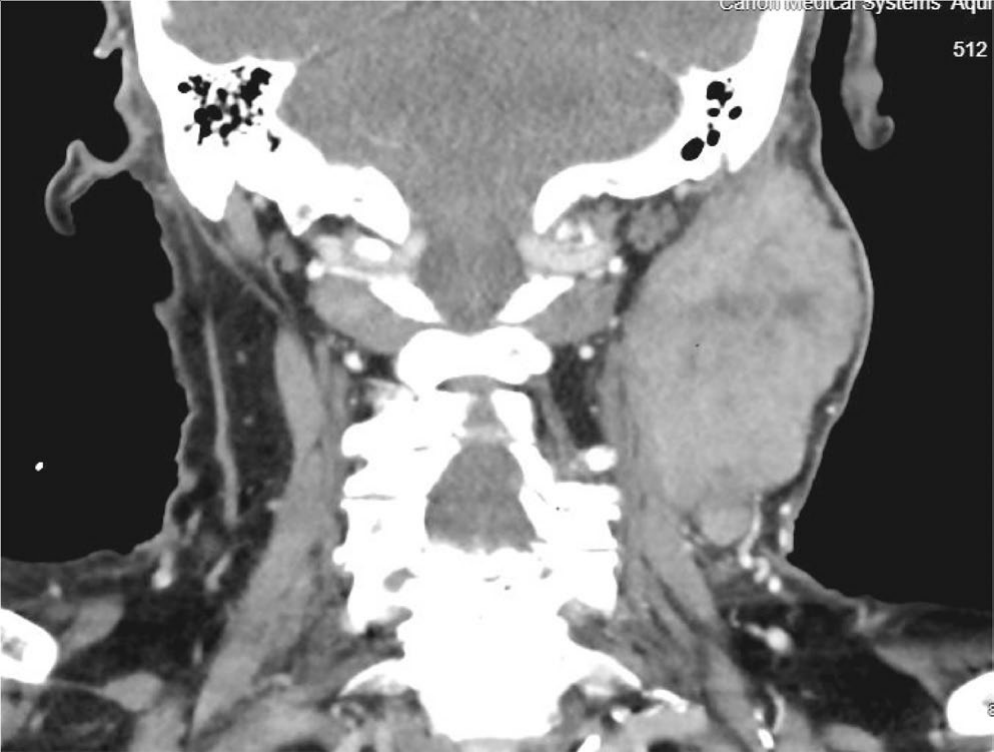
CT neck scan one month prior to clinic presentation.

On examination, there was a large ulcerating and fungating neck mass, extending superficially along the parotid gland and levels 2 and 3. CT scan organized prior to outpatient review showed a mass centred on the left parotid gland measuring 7.9 × 4.5 × 7.7 cm (Figure 1).

**FIGURE 2:**
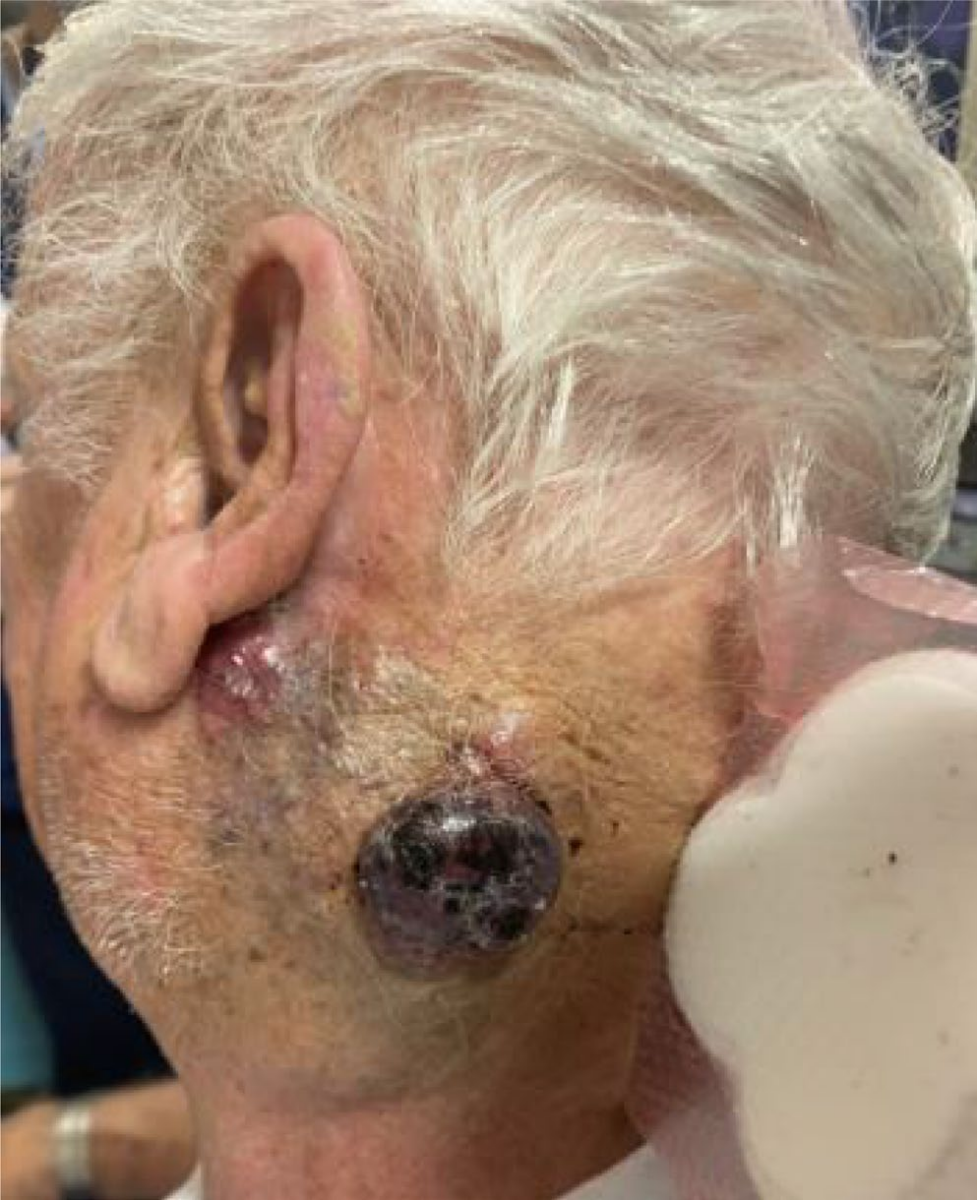
Clinical photograph prior to commencing immunotherapy.

**FIGURE 3:**
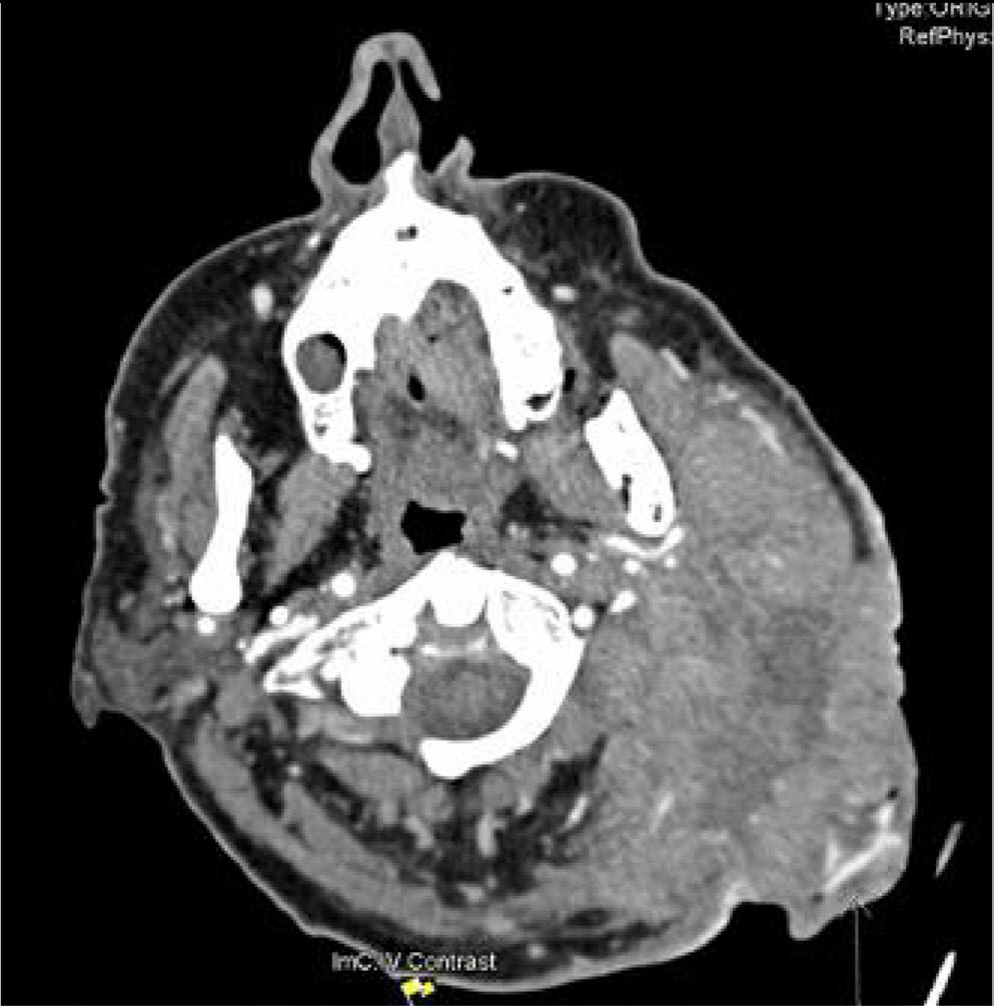
CT neck prior to commencing immunotherapy.

On second presentation to outpatient clinic one month later the mass was clinically deemed to have at least doubled in size. (Figure 2) CT performed two months following clinic presentation showed an increase in size to 10.8 cm. (Figure 3) Fine needle aspirate of the tumor was performed, showing small cell carcinoma morphology, which combined with staining, was shown to be consistent with MCC.

## 4 Treatment

**FIGURE 4:**
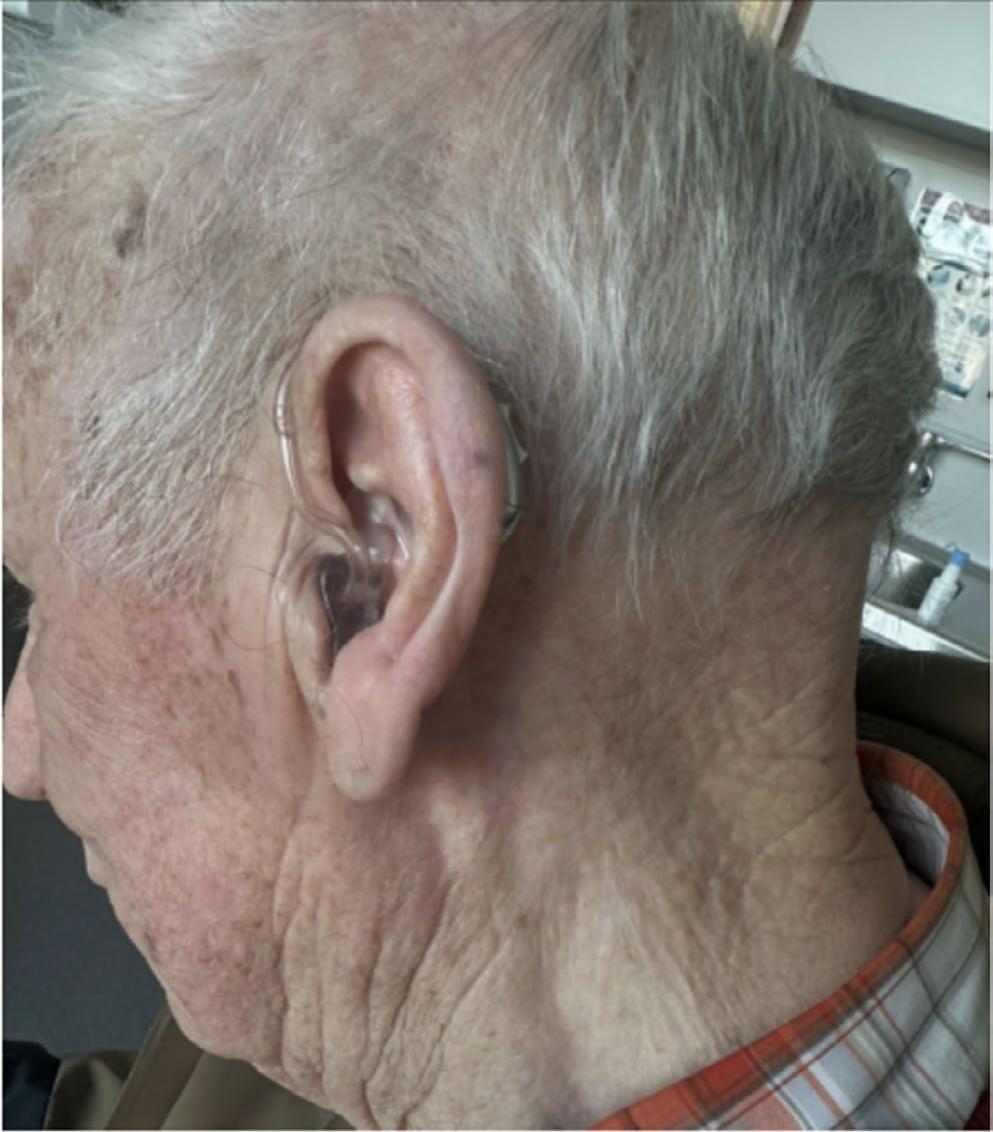
Clinical photograph highlighting complete clinical response to treatment.

Through Multidisciplinary head and neck oncology meeting he was referred to medical oncology for immunotherapy for treatment of stage IIIB MCC [5]. This was with palliative intent, as he was deemed unsuitable for surgical intervention. He was started on Avelumab 800 mg, given fortnightly, and he continues to receive this immunotherapy treatment. He was also referred to radiation oncology with the aim of control of symptomatic bleeding from the tumor. He received a 60 gray course of radiotherapy over 6 sessions for symptomatic relief. The patient had an immediate response to Avelumab and was deemed to have a complete clinical response on clinical examination 4 months after starting treatment, with no palpable mass or lymphadenopathy appreciable on examination. (Figure 4) A complete pathological response could not be confirmed given the patient had not been planned for any surgical intervention.

**FIGURE 5:**
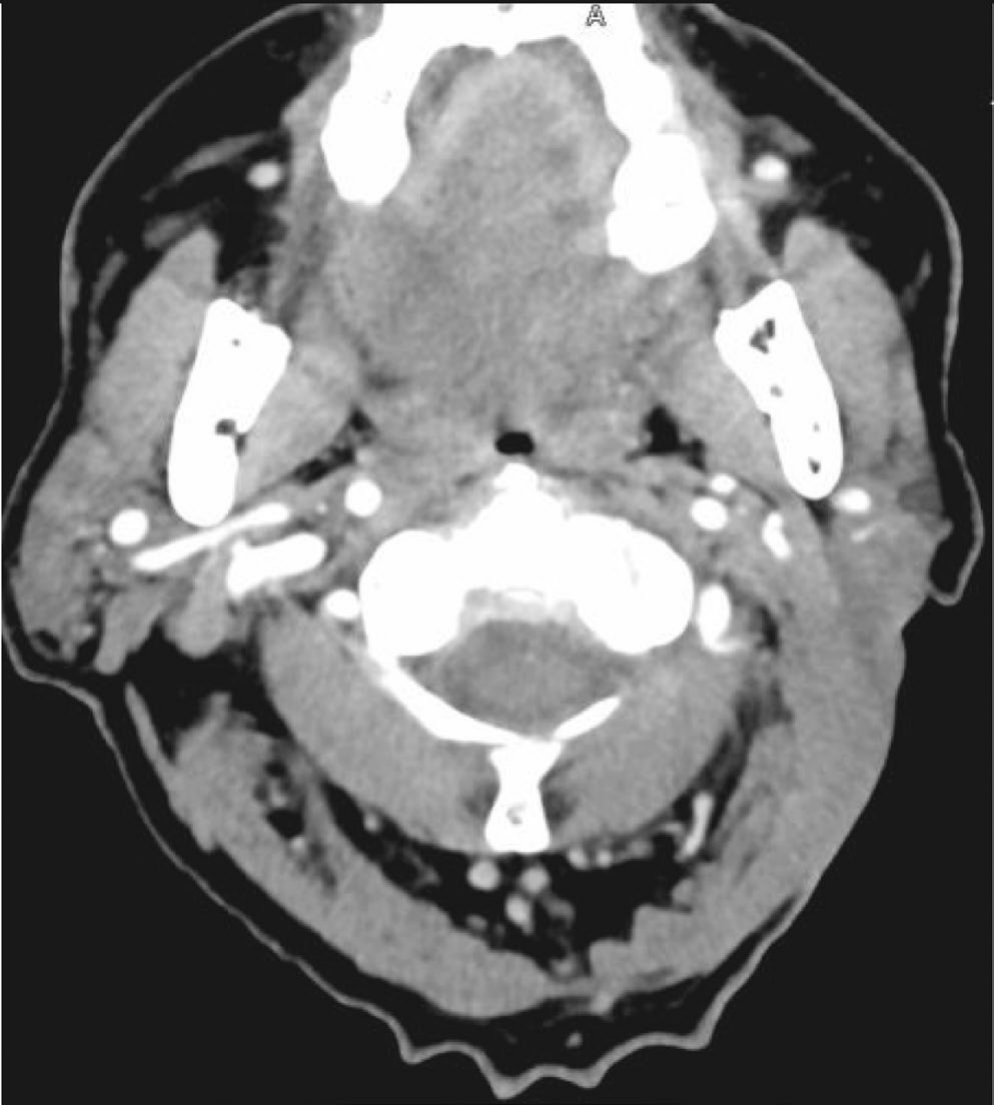
CT neck at 12 months post initiation of treatment highlighting complete radiological response to treatment.

Scans performed at 3 months post immunotherapy showed no evidence of residual disease, a complete radiological response. This has persisted with CT neck scans performed at 12 months. (Figure 5) The patient has avoided surgical intervention and is continuing maintenance immunotherapy with Avelumab. He is being followed up 3 monthly, both radiologically and clinically, in otolaryngology and oncology clinic.

## 5 Results and Conclusion

On presentation this 85 year‐old gentleman had a stage IIIB MCC for which immunotherapy was given as first line treatment, with concurrent palliative radiotherapy for symptom management. Within one month of immunotherapy he exhibited a remarkable response, with a complete radiological response seen at three months following treatment. He continues to show no signs of residual or recurrent disease without any adverse effects of his immunotherapy.

A complete response can be defined as disappearance of all signs of cancer [12]. In this case, the patient had no clinical evidence of malignant disease. In pathological terms, a lack of cancer cells in tissue samples post treatment denotes a complete pathological response [13]. Without surgical intervention or biopsy, that could not be confirmed in this case.

The use of Avelumab in the treatment of MCC has grown over the last number of years. Clinical trials such as the JAVELIN trial have highlighted its effectiveness as first‐line treatment in those with advanced or locally advanced MCC [14]. Further research is needed into the long term recurrence free survival rate in those on Avelumab, given 46.6% of patients experience disease progression once ceasing immune checkpoint inhibitors, at a median of 11.3 months following treatment cessation. However, those who discontinue immune checkpoint inhibitors after a period of having a complete response to treatment have lower rates of disease progress compared to those who had a non‐complete response (23.8% vs. 66.7%) [15].

Increased use of immunotherapy in head and neck cancer has yielded promising results, with complete responses reported in mucosal head and neck cancers including squamous cell carcinoma [16].

## 6 Discussion

Our case demonstrates a sustained clinical response to immunotherapy for MCC in a patient who may have been considered for palliative treatment options only. It is limited being a single case study, coupled with the fact that no pathological complete response has been confirmed. With further research, we believe the indications for immunotherapy will broaden both as a monotherapy and in combination with other management strategies, such as alongside radiotherapy as reported in this case.

## Author Contributions

**Eamonn Byrne:** conceptualization, data curation, writing – original draft. **Mel Corbett:** data curation, writing – review and editing. **Eoin Conlon:** data curation, writing – original draft. **Fergal O'Duffy:** supervision, writing – review and editing.

## Funding

The authors have nothing to report.

## Ethics Statement

Our institution does not require ethics approval for reporting of individual cases.

## Consent

Written informed consent was obtained from the patient for the use of photographs and production of the case report.

## Conflicts of Interest

The authors declare no conflicts of interest.

## Data Availability

The authors confirm that the data in relation to the case report are available within the publication, or from author EB on request.
